# Association of Dietary intake of vitamin E with chronic obstructive pulmonary disease events in US adults: A cross-sectional study of NHANES 2013–2018

**DOI:** 10.3389/fnut.2023.1124648

**Published:** 2023-04-12

**Authors:** Ziyi Liu, Yingjie Su, Qing Chen, Lihua Xiao, Xue Zhao, Feichi Wang, Zhenyu Peng, Hongliang Zhang

**Affiliations:** ^1^Department of Emergency Medicine, Second Xiangya Hospital, Central South University, Changsha, Hunan, China; ^2^College of Medicine, Hunan Normal University, Changsha, China

**Keywords:** vitamin E, COPD, subgroups, cross-sectional studies, NHANES

## Abstract

**Introduction:**

Several studies have demonstrated that vitamin E intake is negatively associated with the development of several diseases, but the relationship between vitamin E intake and COPD in different groups of people is not clear. The aim was to investigate the relationship between vitamin E intake and COPD in different groups of people.

**Methods:**

This study used data from NHANES (National Health and Nutrition Examination Survey) from 2013–2018. A final total of 4,706 participants were included, univariate versus multivariate logistic regression and restricted cubic spline models adjusted for confounders were used to explore the relationship between vitamin E intake and COPD, and subgroup analyses were conducted to assess whether there are differences in the relationship between vitamin E intake and COPD in different populations or conditions.

**Results:**

After adjusting for potential confounders, higher vitamin E intake showed a significant negative association with COPD [Model 1(unadjusted covariates, OR = 0.48;95% CI:0.33–0.70; *p* < 0.001), Model 2(adjusted for age, sex, and race, OR = 0.48;95% CI:0.31–0.73; *p* < 0.01), and Model 3(adjusted for all covariates, OR = 0.57;95% CI:0.36–0.91; *p* = 0.02)]. And a restricted cubic spline curve showed a significant negative correlation between vitamin E intake and COPD (*p* for nonlinear = 0.2036). In the subgroup analysis, we found a negative association between vitamin E intake and COPD in all subgroups as well.

**Conclusion:**

After analyzing data based on the NHANES database from 2013–2018, the results showed that vitamin E intake among U.S. adults was well below the recommended levels and that higher vitamin E intake was negatively associated with COPD incidence.

## 1. Introduction

Chronic obstructive pulmonary disease (COPD) is a lifelong respiratory disease that results in permanently impaired respiratory function from which there is no recovery (1). By 2030, COPD will rank as the third-leading global cause of death and the fourth-leading cause of death in the United States, according to the World Health Organization. The disease burden of COPD is expected to rise in the coming decades (2, 3). Its high prevalence, morbidity, and mortality pose a great challenge to the world’s healthcare systems and are a major global public health problem (4, 5). Therefore, the search for factors that can prevent and treat the development of COPD is extremely necessary. Several studies have shown that dietary factors are associated with the development of COPD, and the risk of COPD is positively associated with the intake of green tea, soy foods, and dietary fiber, and negatively associated with soda, coffee, and vitamin D intake (6–9). Vitamin E is a vitamin with an antioxidant function and one of its main functions is to prevent the peroxidation of lipid molecules. And since oxidative stress is one of the important features of COPD, vitamin E may be a factor in the prevention of COPD (10). In respiratory diseases, several cohort studies have revealed the benefits of vitamin E in reducing the risk of respiratory diseases such as asthma, lung cancer, and upper respiratory tract infections (11–13). A large, randomized trial showed that increased vitamin E intake significantly reduced the risk of developing chronic obstructive pulmonary disease in women (14). However, studies on the association between vitamin E and COPD disease prevalence in large samples of the US population are extremely limited and, as mentioned above, there are limitations in considering only females and not replicating the study across age groups, education levels, marital status, and ethnicity. Therefore, based on surveillance data from the National Health and Nutrition Examination Survey (NHANES) from 2013 to 2018, we carried out a significant cross-sectional study to investigate the relationship between vitamin E intake and COPD, considering various age groups, genders, educational levels, marital status, and ethnicity. We predicted that a higher intake of vitamin E would be linked to a decreased incidence of COPD.

## 2. Materials and methods

### 2.1. Study population

**Figure 1:**
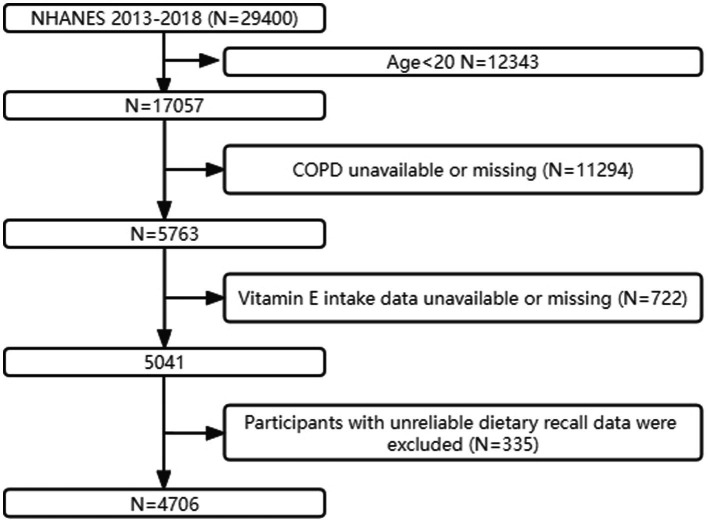
Flow chart of participant screening based on the NHANES database for the relationship between COPD and VE from 2013–2018. COPD: chronic obstructive pulmonary disease.

We obtained data from the NHANES database for the 2013–2018 survey cycle for the analysis of a cross-sectional study, and we excluded participants aged less than 20 years (*N* = 12,324). Individuals lacking information on chronic obstructive pulmonary disease (COPD) status with unreliable information (*N* = 11,294) were also excluded. In addition, participants with missing information on vitamin E intake (*N* = 722) were excluded, as were those with daily vitamin E intake above the mean + 3 SD or below the mean − 3 SD or missing diet weight (*N* = 335), resulting in a total of 4,706 participants being included in the final analysis (Figure 1). The National Health Statistics Research Ethics Review Board authorized the study protocol. Before the interview and assessment, each subject gave their free and informed consent. The National Health and Nutrition Examination Study is a cross-sectional population-based survey created to gather data on the health and nutrition of the population of American households (15). The Institutional Review Board of the Centers for Disease Control also gave its approval to the study protocol. All subjects included in the study provided written informed consent.

### 2.2. Definition of independent and dependent variables

By examining the medical status questionnaire from self-reported personal interview data on various health issues in children and adults supplied in the medical status portion of the NHANES database, we were able to determine the outcomes of the dependent variable. A computer-assisted personal interview (CAPI) technology was used by trained interviewers to conduct the questions at their homes. On the website of the National Institutes of Health, the surveys are accessible. When asked if a doctor or other healthcare provider had informed her or him that they had COPD. And numerous peer-reviewed studies have demonstrated the validity of the self-reported method for diagnosing COPD (16–19). When he or she answered yes, we considered him or her to have COPD. We collected detailed information on dietary intake from participants using the dietary interview section of the NHANES database. The first dietary recall interview was collected in person at the Mobile Examination Center (MEC), and this study defined the first 24-h vitamin E intake as the independent variable (20).

### 2.3. Covariates

A standard questionnaire was used to collect demographic information including age (20–40 years, 40–60 years, or older than 60 years), gender (male or female), race (Mexican American, non-Hispanic white, non-Hispanic black, or other race), marital status (married/cohabiting with a partner, widowed/divorced/separated, or never married), an education level (less than high school, high school or greater than high school). Other confounders included: Smoking was defined as consuming at least 100 cigarettes in a lifetime, and hypertension is defined as a mean systolic blood pressure greater than 140 mmHg or a mean diastolic blood pressure greater than 90 mmHg or a self-reported physician’s diagnosis (21, 22). Participants with fasting blood glucose levels above 126 mg/dL or 2-h blood glucose levels above 200 mg/dl (measured by oral glucose tolerance test) or treated with insulin or anti-diabetic medication were considered to have diabetes (23). BMI is calculated by dividing body weight (kg) by height squared (m2). Standard biochemical techniques were employed in the NHANES database to assess the amounts of total cholesterol, triglycerides, low-density lipoprotein cholesterol (LDL), high-density lipoprotein cholesterol (HDL), and glycated hemoglobin. The Laboratory Methods Documentation portion of the NHANES contains a thorough overview of the lab procedures performed. Random forest interpolation was performed for covariates with missing values (24).

### 2.4. Statistical analyses

NHANES uses a complex multistage probability sampling design and therefore uses appropriate weighting in our statistical analyses. In the baseline information, continuous variables were expressed as mean ± standard deviation, and categorical variables were expressed as percentages. Weighted ANOVA and weighted chi-square tests were used to compare between-group differences for continuous and categorical variables, respectively. Univariate logistic regression analysis and multivariate logistic regression analysis were used to explore the relationship between vitamin E intake and the incidence of COPD events. Dietary vitamin E intake was categorized into tertiles. To adjust for the effects of confounding factors, we performed model adjustments, Model 1: no adjustment, Model 2: adjusted for demographic information such as age, gender, and race, and Model 3: adjusted for information on all covariates (age, gender, race, marriage, education level, BMI, HDL, triglycerides, glycohemoglobin, LDL, smoking status, diabetes, hypertension, and vitamin E intake). We used a restricted cubic spline curve for describing the dose–response relationship between vitamin E intake and COPD and plotted dose–response curves to visualize the association between vitamin E intake and the incidence of COPD events. We also performed subgroup analyses (grouped by age, sex, race, marital status, education level, smoking status, hypertension, and diabetes). The aim was to discuss the stability of the association between vitamin E intake and the incidence of incident COPD in different subgroups, to discuss the interaction between different covariates by likelihood ratio tests, and to visualize the results as forest plots for subgroup analysis. We performed all analyses by using R version 4.21 (25–27).

## 3. Results

### 3.1. Baseline data characteristics

A total of 29,400 participants were enrolled in this study, and a total of 4,706 participants were recruited for this study, of whom 47.7% were female, 52.3% were male, 9.1% were Mexican American, 65.4% were non-Hispanic white, 11.3% were non-Hispanic black, and 14.3% were of other races. In addition, 62.1, 18.9, and 19.0% of participants were married/living with a partner, widowed/divorced/separated, and never married, respectively. In addition, 63.3% have a degree higher than high school, 21.9% had only a high school education, and 19.0 percent had an education below high school. In addition, 43.6% smoked, 14.1% had diabetes, and 40.1% had hypertension. A weighted population baseline table was derived based on the presence or absence of COPD (Table 1). The largest proportion of patients with COPD in this study was aged >60 years, 51.3% were male and 48.7% were female, and the proportion of non-Hispanic white (84.8%) was much higher than the rest of the population. For the factors of interest, we found a significantly higher intake of vitamin E in those without COPD (8.72 mg/day) than in those with COPD (7.10 mg/day).

**Table 1:** Baseline information, weighted.

Characteristic	COPD			
	Overall	No	Yes	*p* value
*N*	4706	4551	155	
*Age, n (%)*				<0.001
20–40 years	37.5	38.8	3.1	
40–60 years	37.5	37.3	41.7	
>60 years	25.0	23.9	55.2	
*Gender, n (%)*				
Male	52.3	52.4	51.3	0.805
Female	47.7	47.6	48.7	
*Race, n (%)*				0.010
Mexican america	9.1	9.4	2.8	
Other races	14.3	14.5	7.1	
Non-hispanic white	65.4	64.6	84.8	
Non-hispanic black	11.3	11.5	5.2	
*Marriage, n (%)*				<0.001
Married/living with a partner	62.1	62.5	52.1	
Widowed/divorced/separated	18.9	18.0	42.5	
Never married	19.0	19.5	5.4	
*Education, n (%)*				0.004
<High school	14.8	14.3	26.4	
High school	21.9	21.7	29.3	
>High school	63.3	64.0	44.2	
*BMI, kg/m^2^*	29.22 ± 7.16	29.19 ± 7.12	30.05 ± 8.19	0.321
*Hdl, mg/dL*	53.24 ± 16.31	53.35 ± 16.21	50.30 ± 18.59	0.155
*TC, mg/dL*	190.77 ± 41.36	190.95 ± 41.26	185.88 ± 43.90	0.361
*Triglyceride, mg/dL*	124.95 ± 90.04	123.73 ± 89.26	157.74 ± 103.98	0.012
*LDL, mg/dL*	113.11 ± 29.58	113.36 ± 29.57	106.45 ± 29.36	0.064
*Glycohemoglobin (%)*	5.62 ± 0.95	5.60 ± 0.94	6.03 ± 1.14	0.001
*VEintake, mg/day*	8.66 ± 5.52	8.72 ± 5.55	7.10 ± 4.39	<0.001
*Smoking status, n (%)*				<0.001
No	56.4	57.9	15.7	
Yes	43.6	42.1	84.3	
*Diabetes, n (%)*				<0.001
No	85.9	86.7	64.5	
Yes	14.1	13.3	35.3	
*Hypertension, n (%)*				<0.001
No	59.9	60.9	31.3	
Yes	40.1	39.1	68.2	

Mean ± standard error (SE) for continuous variables, percentages (%) for categorical variables. BMI, body mass index; HDL, high-density lipoprotein; TC, Total cholesterol; LDL, low-density lipoprotein; VEintake; vitamin E intake； COPD: chronic obstructive pulmonary disease.

### 3.2. Higher vitamin E intake is associated with a lower incidence of COPD events

**Figure 2:**
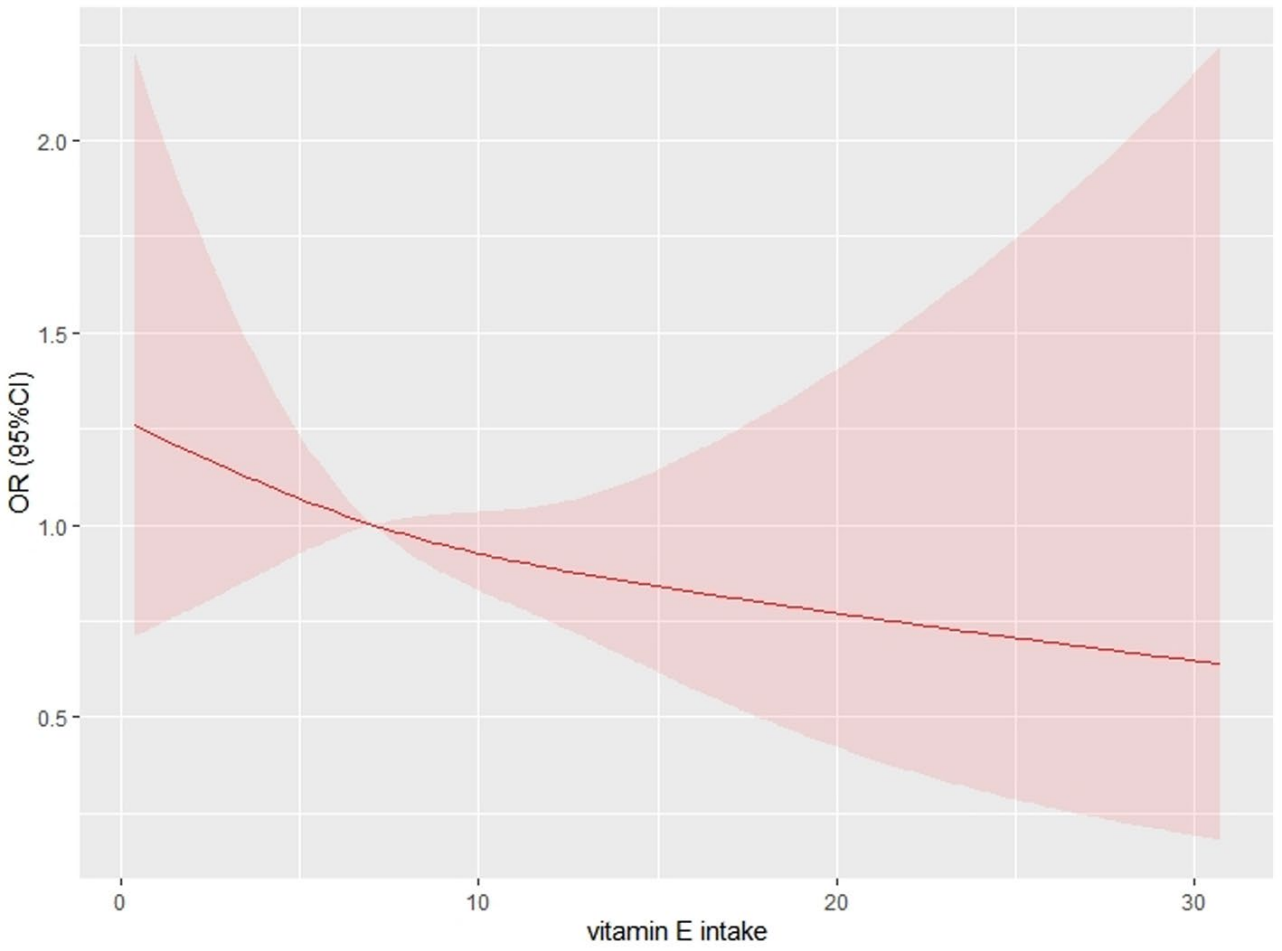
Restricted cubic spline curve for describing the dose–response relationship between vitamin E intake and chronic obstructive pulmonary disease. The following confounding variables were adjusted for (age; sex; race; marital status; education level; BMI; HDL; total cholesterol; total triglycerides; smoking status; glycosylated hemoglobin level; diabetes mellitus; hypertension;).

**Figure 3:**
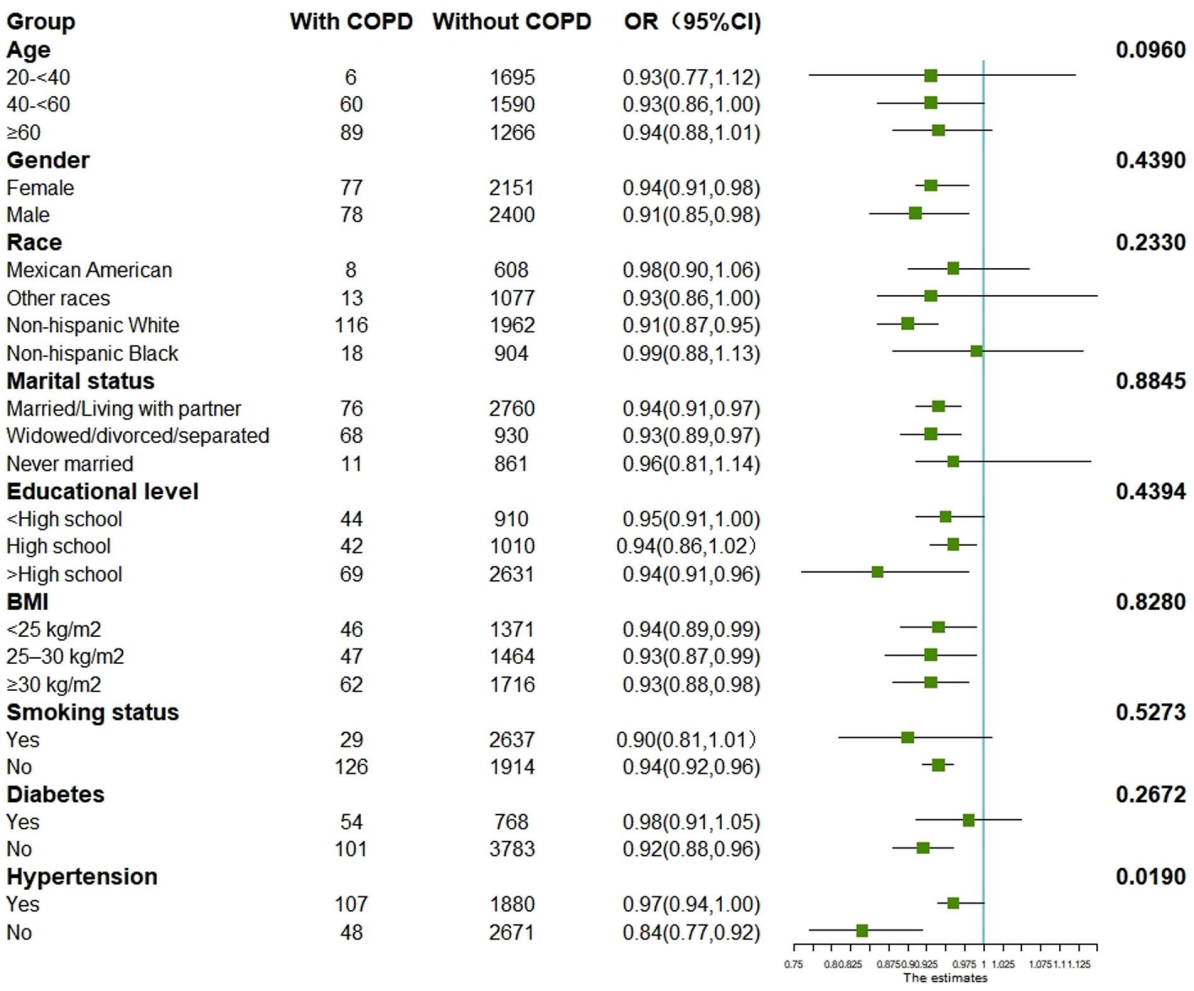
Forest plots for subgroup analysis.

We performed univariate logistic regression analysis, and the results are shown in Table 2. From Table 2 we can find that the second tertile (OR = 0.83; 95% CI, 0.46–1.50), and the highest tertile (OR = 0.48; 95% CI, 0.33–0.70) reduced COPD compared to the lowest tertile, in addition, Table 3 presents the results of multivariate logistic regression between vitamin E intake and COPD for model 1(unadjusted covariates, OR = 0.48;95% CI:0.33–0.70; *p* < 0.001), model 2(adjusted for age, sex, and race, OR = 0.48;95% CI:0.31–0.73; *p* < 0.01), and model 3(adjusted for all covariates, OR = 0.57;95% CI:0.36–0.91; *p* = 0.02). The highest tertile vitamin E intake was significantly negatively associated with COPD compared with the lowest tertile. Finally, we plotted the dose–response relationship between vitamin E intake and COPD events based on the restrictive cubic curve and found a strong linear relationship (*p* for nonlinear = 0.2036). We removed data defined as vitamin E intake that was 3 standard deviations from the mean, which effectively avoided the effect of extreme data on the statistical analysis. As shown in Figure 2, vitamin E intake was negatively associated with COPD events. In addition, we performed a subgroup analysis (Table 4) and visualized (Figure 3) the results indicating that the negative association between vitamin E intake and COPD incidence is robust. In conclusion, higher vitamin E intake was consistently and negatively associated with COPD events.

**Table 2:** Weighting the results of the one-way logistic regression analysis for the factors associated with COPD.

	OR	*p* value
Gender		
Female	Ref	
Male	0.95 (0.66,1.37)	0.805
Race		
Mexican american	Ref	
Other races	1.63 (0.30,8.79)	0.545
Non-his panic white	4.35 (1.60,11.81)	0.006
Non-his panic black	1.51 (0.52,4.42)	0.418
Marriage		
Married/Living with partner	Ref	
Widowed/divorced/separated	2.83 (1.86,4.31)	<0.001
Never married	0.33 (0.14,0.78)	0.014
Education		
< High school	Ref	
High school	0.73 (0.43,1.24)	0.234
>High school	0.37 (0.19,0.70)	0.004
BMI	1.01 (0.98,1.04)	0.289
HDL	0.98 (0.96,1.00)	0.191
TC	0.99 (0.98,1.00)	0.371
Triglyceride	1.00 (0.99,1.00)	0.232
LDL	0.99 (0.98,1.00)	0.031
Glycohemoglobin	1.31 (1.17,1.48)	<0.001
Smoking status		
No	Ref	
Yes	6.91 (3.91,12.20)	<0.001
Diabetes		
No	Ref	
Yes	3.55 (2.29,5.51)	<0.001
Hypertension		
No	Ref	
Yes	3.34 (2.29,4.85)	<0.001
Age		
20–40 years	Ref	
40–60 years	14.02 (4.71,41.70)	<0.001
>60 years	28.93 (10.10,82.89)	<0.001
VEintake		
Q1	Ref	
Q2	0.83 (0.46,1.50)	0.523
Q3	0.48 (0.33,0.70)	<0.001

*p*-Value was calculated via logistic regression analysis. Q1 (≤5.370 mg/day), Q2 (5.370 to 9.230 mg/day), Q3 (≥9.230 mg/day). OR, odds ratio; 95%CI, 95% confidence interval. BMI, body mass index; HDL, high-density lipoprotein; TC, Total cholesterol, LDL, low-density lipoprotein; VEintake; vitamin E intake.

**Table 3:** Results of multiple logistic regression analysis of the association between vitamin E intake and COPD, weighted.

	Model 1		Model 2		Model 3	
	OR (95%CI)	*p* value	OR (95%CI)	*p* value	OR (95%CI)	*p* value
**Q1**	Ref	Ref	Ref	Ref	Ref	Ref
**Q2**	0.83 (0.46,1.50)	0.52	0.84 (0.44,1.59)	0.57	0.89 (0.47,1.68)	0.71
**Q3**	0.48 (0.33,0.70)	<0.001	0.48 (0.31,0.73)	<0.01	0.57 (0.36,0.91)	0.02

OR, odds ratio; 95% CI, 95% confidence interval. Q1 (≤5.370 mg/day), Q2 (5.370 to 9.230 mg/day), Q3 (≥9.230 mg/day).

Model 1: Adjusted for no covariates.

Model 2: Adjusts for basic information such as age, ethnicity, and gender.

Model 3: Adjust for all confounding factors.

## 4. Discussion

In this cross-sectional investigation, 4,706 people were examined, and patients with COPD received significantly less vitamin E on average (7.10 mg daily) than those without COPD (8.72 mg daily). Higher vitamin E intake was found to be independently linked with decreased COPD prevalence after controlling for variables (age, sex, race, marital status, education level, smoking status, consumption status, hypertensive disease, and diabetes) in multivariate logistic regression analysis. By charting a restricted cubic spline curve, we discovered a linear association between vitamin E intake and COPD, with a tendency for the incidence of COPD to decline with higher vitamin E intake. Additionally, our research revealed that the study population’s average daily consumption of total vitamin E (8.66 mg) was much lower than the recommended level (15 mg). Thus, supplementation of vitamin E intake appears to have an important role in the prevention of COPD.

**Table 4:** Sub-group analysis.

	OR (95%CI)	*p* for interaction
*Stratified by Age*		0.0960
Age20-40	0.93 (0.77,1.12)	
Age40-60	0.93 (0.86,1.00)	
Age60-100	0.94 (0.88,1.01)	
*Stratified by gender*		0.4390
Female	0.94 (0.91,0.98)	
Male	0.91 (0.85,0.98)	
*Stratified by race*		0.2330
Mexican America	0.98 (0.90,1.06)	
Other races	0.93 (0.86,1.00)	
Non-hispanic White	0.91 (0.87,0.95)	
Non-hispanic Black	0.99 (0.88,1.13)	
*Stratified by Marital status*		0.8845
Married/Living with partner	0.94 (0.91,0.97)	
Widowed/divorced/separated	0.93 (0.89,0.97)	
Never married	0.96 (0.81,1.14)	
*Stratified by Educational level*		0.4394
< High school	0.95 (0.91,1.00)	
High school	0.94 (0.86,1.02)	
>High school	0.94 (0.91,0.96)	
*Stratified by BMI*		0.8280
<25 kg/m^2^	0.94 (0.89,0.99)	
25–30 kg/m^2^	0.93 (0.87,0.99)	
≥30 kg/m^2^	0.93 (0.88,0.98)	
*Stratified by smoking status*		0.5273
Yes	0.94 (0.92,0.96)	
No	0.90 (0.81,1.01)	
*Stratified by diabetes*		0.2672
Yes	0.98 (0.91,1.05)	
No	0.92 (0.88,0.96)	
*Stratified by hypertension*		0.0190
Yes	0.97 (0.94,1.00)	
No	0.84 (0.77,0.92)	

Subgroup analyses were performed on the following covariates (age, sex, race, marital status, education level, body mass index; smoking status; alcohol consumption status; diabetes mellitus; hypertension. OR, odds ratio; 95% CI, 95% confidence interval. BMI, body mass index; HDL, high-density lipoprotein; TC, total cholesterol; LDL, low-density lipoprotein; VE intake; vitamin E Intake).

Numerous research has looked at the connection between vitamin E intake and COPD, and one of those studies found a link between lung function and vitamin E intake (10). In contrast, two other studies (28, 29) reported no independent effect of vitamin E intake after correction but a significant correlation between lung function and vitamin E intake when confounders were taken into account before adjustment. There is no conclusive evidence linking vitamin E and lung health. These discrepancies may be the result of insufficient sample sizes as well as variations in the dietary cultures, ethnicities, etc. of the populations under study. A significant cohort follow-up study showed a negative association between vitamin E intake and COPD incidence (14).

The underlying biological mechanisms for this association are complex and not yet clear. The deterioration of the lung parenchyma and persistent inflammation of the small airways are two symptoms of COPD, according to several studies (30, 31). Oxidative stress in the lung is one of the main mechanisms of its pathogenesis (31, 32). The endogenous antioxidant function is reduced by the presence of reactive oxygen species (ROS), which becomes a cause of oxidative stress (33). Vitamin E, on the other hand, is characterized by having an antioxidant function. Several studies have shown that supplemental vitamin E intake increases the elevation of antioxidants in the body and is effective in improving indicators of lung function (10, 34). One study showed that Trolox (a water-soluble derivative of vitamin E) was able to prevent oxidative stress, genotoxicity, and inflammation in the lung through a mechanism of ROS scavenging (35). Inhibiting the EGFR/MAPK axis lowers inflammation, apoptosis, and ROS, which inhibits COX2-mediated p-STAT3 nuclear translocation and lessens symptoms of COPD, according to recent research on vitamin E intake (36).

Our study has several benefits. To ensure the validity of the results, we used the proper weights and confounder adjustments during the analysis. Second, only a few studies have looked at the relationship between vitamin E and the incidence of COPD in large samples; our study is a big sample study using the NHANES database. Third, our study is the first to examine the relationship between vitamin E and COPD across ethnicity and gender in the United States and to assess the validity of the findings across ethnicity and gender, whereas earlier studies have only examined specific populations, such as women.

However, limitations are unavoidable. To start, because this study was cross-sectional, it was not possible to determine the cause of the link between vitamin E and COPD. Second, a 24-h recall may not be a reliable basis for measuring vitamin E intake. Additionally, because the data were self-reported, which has been demonstrated in various research to overstate the incidence of COPD, there may have been a bias introduced.

## 5. Conclusion

After analyzing data based on the NHANES database from 2013–2018, the results showed that vitamin E intake among U.S. adults was well below the recommended levels and that higher vitamin E intake was negatively associated with COPD incidence.

## Data availability statement

The raw data supporting the conclusions of this article will be made available by the authors, without undue reservation.

## Ethics statement

The studies involving human participants were reviewed and approved by National Center for Health Statistics- United States. The patients/participants provided their written informed consent to participate in this study.

## Author contributions

ZL, YS, and FW: conceptualization and formal analysis. XZ, LX, and QC: methodology, software, and survey. ZL and HZ: data collation and writing-original draft preparation. HZ and ZP: writing-review and editing and supervision. HZ was the guarantor of this work, and as such, had full access to all the data in the study and assumes responsibility for the integrity of the data and the accuracy of the data analysis. All authors contributed to the article and approved the submitted version.

## Conflict of interest

The authors declare that the research was conducted in the absence of any commercial or financial relationships that could be construed as a potential conflict of interest.

## Publisher’s note

All claims expressed in this article are solely those of the authors and do not necessarily represent those of their affiliated organizations, or those of the publisher, the editors and the reviewers. Any product that may be evaluated in this article, or claim that may be made by its manufacturer, is not guaranteed or endorsed by the publisher.
